# Work and Family Conflicts, Depression, and “Ikigai”: A Mediation Analysis in a Cross-cultural Study Between Japanese and Egyptian Civil Workers

**DOI:** 10.2188/jea.JE20210338

**Published:** 2023-07-05

**Authors:** Ehab S. Eshak, Sachiko Baba, Hiroshi Yatsuya, Hiroyasu Iso, Yoshihisa Hirakawa, Eman M. Mahfouz, Chiang Chifa, Ryoto Sakaniwa, Ayman S. El-khateeb

**Affiliations:** 1 Public Health Department, Faculty of Medicine, Minia University, El-Minia, Egypt; 2 Public Health, Department of Social Medicine, Graduate School of Medicine, Osaka University, Osaka, Japan; 3 Bioethics and Public Policy, Department of Social Medicine, Osaka University Graduate School of Medicine, Osaka, Japan; 4 Department of Public Health and Health System, Graduate School of Medicine, Nagoya University, Nagoya, Japan

**Keywords:** cross-cultural study, work-family conflict, depression, ikigai, civil workers, Japan, Egypt

## Abstract

**Background:**

Total work-family conflicts (TWFCs) could associate with mental health, and having ikigai (a purpose of life) may mediate this association.

**Methods:**

In a cross-cultural study of 4,792 Japanese Aichi Workers’ Cohort study participants and 3,109 Egyptian civil workers, the Midlife Development in the United States (MIDUS) questionnaire measured TWFCs and the Center for Epidemiological Studies Depression (CES-D) 11-item scale measured depression. We used logistic regression models to estimate odds ratios (ORs) of having depression and a high-ikigai across levels of TWFCs (low, moderate, and high), and the PROCESS macro of Hayes to test the mediation effect.

**Results:**

The prevalence of high TWFCs, depression, and having a high ikigai were 17.9%, 39.4%, and 70.1% in Japanese women, 10.5%, 26.8%, and 70.1% in Japanese men, 23.7%, 58.2%, and 24.7% in Egyptian women, and 19.1%, 38.9%, and 36.9% in Egyptian men, respectively. Compared with participants with low TWFCs, the multivariable ORs of depression in Japanese women and men with high TWFCs were 4.11 (95% confidence interval [CI], 2.99–5.65) and 5.42 (95% CI, 4.18–7.02), and those in Egyptian women and men were 4.43 (95% CI, 3.30–5.95) and 4.79 (95% CI, 3.53–6.48), respectively. The respective ORs of having a high-ikigai were 0.46 (95% CI, 0.33–0.64) and 0.40 (95% CI, 0.31–0.52) in Japanese women and men and were 0.34 (95% CI, 0.24–0.48) and 0.28 (95% CI, 0.20–0.39) in Egyptian women and men. No interaction between TWFCs and country was observed for the associations with depression or ikigai. Ikigai mediated (up to 18%) the associations between the TWFCs and depression, especially in Egyptian civil workers.

**Conclusion:**

TWFCs were associated with depression, and having low ikigai mediated these associations in Japanese and Egyptian civil workers.

## INTRODUCTION

At least half of the leading causes of disability worldwide are psychological and mental health problems.^1^ Among these disorders, depression has a definitive impact on any working population.^2^ A high prevalence of depression has been observed in many working populations all around the globe. A study from the 1990s had estimated the lifetime prevalence of major depressive disorders in the Japanese working population as 14%,^3^ while a 5.7% prevalence was estimated between 2013 and 2015 according to the results of the 2nd survey of the World Mental Health Japan.^4^ In the context of another population, the estimated prevalence of depressive symptoms in Egyptians of working age ranged from 23.2% to 37.5%.^5^^,^^6^

The environments at both work^7^ and family^8^ were associated with civil workers’ psychological health. The competing relation between the demands of work and family on individuals’ time, energy, and interests could lead to work-to-family conflict (WFC) or family-to-work conflict (FWC). These conflicts sum to the total work-family conflicts (TWFCs),^9^ which were defined as “forms of inter-role conflicts in which the role pressures from the work and family domains are mutually incompatible in some respect.”^10^ Previous studies documented positive associations between TWFCs and psychological health among American,^11^^–^^14^ European,^15^^,^^16^ Asian,^15^^,^^17^^,^^18^ and Egyptian^19^ working populations. Meanwhile, the work environment for civil workers could differ from one country to another, and the cultural differences in family attributes could also impact the shape and magnitude of the associations between psychological well-being and conflicts arising between work and family.

On the other hand, having ikigai, a Japanese spiritual concept characterized by having a purpose in life and enjoying one’s activities to achieve satisfaction and a sense of meaning, entails the concept of eudaimonic well-being.^20^ In other words, not having ikigai encompasses a state in which one does not have a sense of life purpose. We hypothesized that an ill-balanced work-family life may logically contribute to not having or perceiving ikigai. However, no previous study has investigated the direct association of ikigai with TWFCs or mental health. TWFCs were associated with reduced life satisfaction,^21^ and reduced life satisfaction was associated with depression.^22^ Thus, having a low ikigai may work as a mediator in the association between TWFCs and having depression. Therefore, we compared the associations of TWFCs with the prevalence of having depression and a high ikigai among Japanese and Egyptian civil workers and determined the potential mediating effect of having a low ikigai on the associations between TWFCs and having depression.

## METHODS

### Participants and data collection procedure

The Japanese dataset included 5,543 participants in the Aichi Workers’ Cohort study (a Japanese cohort of civil servants) who responded to the questionnaire distributed in the 2018 data collection cycle. The Egyptian data were based on a convenience sample of 3,143 Egyptian civil workers collected in 2019 by the Department of Public Health at Minia University in Egypt. Details of the Aichi Workers’ Cohort study and the Egyptian survey can be found elsewhere.^23^^,^^24^

For this cross-national study, data were collected using the same self-administered questionnaires to maintain consistency in data gathering procedures in both countries. The questionnaire inquired about civil workers’ lifestyles and medical history. The Egyptian participants provided written informed consent to the comparative study simultaneously while consenting to participate in the Egyptian survey. We offered the Aichi Workers’ Cohort participants a way to opt-out from the comparative study if they wished. Nagoya University in Japan and Minia University in Egypt provided the data for the comparative research to Osaka University, whose ethical review board has approved the comparative, cross-cultural study (approval number 19501/2020).

We limited the participants to those aged 20–60 years old as the retirement in Egypt is at 60 years, which required the age range exclusion of 232 Japanese and 10 Egyptian participants. We further excluded 437 Japanese and 22 Egyptian participants to whom the exposure factor was not applicable or reported being devoid of family members. Also, 82 Japanese and 2 Egyptian participants with missing data on the outcome variables were excluded. Thus, the final sample for the Japanese cohort consisted of 4,792 participants, and that of the Egyptian survey consisted of 3,109 civil workers.

### Total work-family conflicts

The Midlife Development in the United States National Study^25^ validated a three-point Likert scale (0 = never, 1 = to some extent, 2 = often/very often) response to the following four items assessing the FWC: “Some domestic problems reduce the amount of time you can concentrate on your work”, “Home worries or problems can disconcert you from work”, “Housework can prevent you from getting the sleep you need to get your job done”, and “Responsibilities at home reduce the time to relax as yourself”. The four items assessed the WFC were: “Because of my work, I spend less time with my family”, “I am annoyed at home because of problems at work”, “I often leave home on business trips”, and “At home, I think I can’t do anything that requires my attention because my work consumes my energy”, and were similarly scaled. The scores of FWC and WFC ranged from 0–8 points and were used in previous studies conducted in Japanese^26^ and Egyptian^27^^,^^28^ populations and showed good internal consistencies by the Cronbach’s alpha test (TWFCs in Japan: 0.77 for men and 0.79 for women^26^; TWFCs, FWCs, and WFCs in Egypt: 0.87, 0.79, and 0.77 for men and women combined, respectively).^27^^,^^28^

We categorized these conflict scores as low conflict level (<2 points), moderate conflict level (3–4 points), and high conflict level (≥5 points). Participants who were categorized as low in both scores or low in one score and moderate in the other score composed the group of low TWFCs, those who were classified as moderate in both scores or high in one score while low in the other one were categorized as the moderate TWFCs group, while those ranked high in both scores or high in one score and moderate in the other score were classified as the high TWFCs group.

### The outcome and mediator variables

We used the Center for Epidemiological Studies Depression (CES-D) 11-item scale^29^ to assess clinical depression. Each item in the scale was rated on a four-Likert score (0 = rarely to 3 = always). According to a previous study in Japan,^30^ a score ≥8 indicated depression.

Possible responses to the ikigai question, “Do you have ikigai in your life?” in the Japanese survey and “Do you have a purpose of life and feel like enjoying all the activities since life is worth living” in the Egyptian survey, were 1 = very much, 2 = yes, 3 = not so much, 4 = no. The Egyptian participants received verbal explanation to the ikigai question to consider the degree of feeling that life is worth living (ie, one’s mission in life is foreseen to be meaningful) as both hedonic (ie, attaining pleasure and avoiding pain avoidance) and eudaimonic (ie, being a fully functioning person) views of well-being.^20^ Participants who chose “yes” or “very much” were considered to have high ikigai, while those who chose “not so much” or “no” were considered to have low ikigai.

### Hypothesized confounding factors

The questionnaire collected data on the following hypothesized confounding factors: 1) sociodemographic characteristics, including age, education (less than high school, high school, or university and above), occupation (clerk, professional, or worker/technician), and marital status (single, married, divorced, or widowed); 2) family factors, including family structure and living companions, which we categorized as (living alone, living with a spouse only, or living in the multigeneration family), number of family members, and the presence of children below 14 years old in the family; 3) work factors, including job hours per day, time to commute to work, shift work, and working overtime or extra job; 4) behavioral factors, including smoking status (never, ex-, and current smoker), alcohol drinking status in the Japanese cohort only (never, ex-, and current drinker), and physical activity measured in metabolic equivalent of task (METs) unit according to hours spent in sitting, standing, walking, active job, sports, and sleep; and 5) history of chronic diseases, including hypertension, diabetes, dyslipidemia, ischemic heart disease, stroke, kidney diseases, liver diseases, and cancer (yes/no for each).

### Statistical analysis

The data were analyzed using the SAS software version 9.4 (SAS Institute Inc, Cary, NC, USA). The differences in sex-specific characteristics of the Japanese and Egyptian civil workers across the categories of TWFCs were tested using linear and logistic regression analyses adjusted for age. Multivariable logistic regression models that included interaction terms were used to compute the odds ratios (ORs) and 95% confidence intervals (CIs) of the likelihood of having depression and high ikigai across TWFCs categories using the low conflicts category as a reference. The first multivariable model was adjusted for the sociodemographic factors. The second model was adjusted further for the family and work factors. The last model was further adjusted for behavioral and past medical history factors, as described above. We also tested the significance of an interaction (a cross-product) term between the TWFCs and the country of the sample in our models. The associations between the two forms of the TWFCs (FWC and WFC) with having depression and high ikigai were also tested. We conducted stratified analyses by the family structure, shift work, and occupational class for the association between TWFCs with having depression and high ikigai.

Multivariable linear regression analysis was used to predict the slope of the CES-D score by a one-point increment in the scores of TWFCs, WFC, and FWC. To test if the ikigai level mediated the effect of these conflicts on depression, we conducted a mediation analysis with the PROCESS macro version 3.0 in SAS^31^ using the multivariable linear regression models at each step. This approach involved four steps and three separate model fits; in the first linear regression model, the correlation between the exposure (TWFCs, WFC, and FWC continuous scores) and the outcome (CES-D continuous score) was tested, the second model tested the correlation between the exposure and the mediator (the level of ikigai as a continuous variable of 1, 2, 3, and 4 for the increasing levels of ikigai). The third model tested the outcome against both the exposure and the mediator as covariates. Finally, we extracted the total, direct, and indirect effects and calculated percentage mediation by ikigai in the parameters estimates of the CES-D score according to the TWFCs, WFC, and FWC. In all models, we adjusted for all the covariates mentioned above. For the indirect effects, if zero was not included in the 95% CIs of bias-corrected 5,000 times bootstrap test, a significant indirect effect was concluded.

## RESULTS

In Japanese women and men, levels of TWFCs were positively associated with marital status, the number of family members and the number of junior family members below 14 years old, and working overtime or extra work. The positive associations were observed of TWFCs with age, professional occupation, and shift work in women, while in men, a positive association was observed with job hours per day. In Egyptian women and men, levels of TWFCs were positively associated with younger age, job hours per day, shift work, working overtime or extra work, and having a history of chronic diseases. Positive associations were observed with commuting time to work in women, while in men, a positive association was observed with METs units (Table 1).

**Table 1.** Sex- and country-specific civil workers’ characteristics (mean [standard deviation]/proportion)^a^ according to levels of total work-family conflicts

	Japan				Egypt			

	Levels of total work-family conflicts				Levels of total work-family conflicts			

	Low	Moderate	High	*P*-trend^b^	Low	Moderate	High	*P*-trend^b^
**Women**								
Number of subjects, *n*	851	440	282		752	479	383	
Total work-family conflicts score	2.0 (1.5)	6.0 (1.2)	9.6 (1.8)	<0.001	3.5 (2.1)	8.1 (1.1)	11.5 (1.7)	<0.001
Work-to-family conflict score	1.3 (1.2)	3.0 (1.2)	4.5 (1.4)	<0.001	1.9 (1.5)	3.4 (1.6)	6.3 (1.4)	<0.001
Family-to-work conflict score	0.7 (1.1)	3.0 (1.3)	5.2 (1.5)	<0.001	1.6 (1.4)	3.6 (1.5)	5.2 (1.4)	<0.001
Age, years	35.8 (11.6)	41.9 (9.9)	42.5 (8.5)	<0.001	40.3 (10.3)	37.7 (9.8)	35.5 (9.4)	<0.001
High education, %	67.3	56.4	63.2	0.22	65.6	68.9	70.0	0.41
Professional occupation, %	51.0	53.2	63.6	<0.001	63.3	68.5	69.5	0.23
Married, %	42.7	72.9	80.6	<0.001	77.4	78.9	77.8	0.33
Living alone, %	13.5	10.5	4.5	<0.001	0.5	0.4	0.5	0.60
Number of family members	2.0 (1.4)	2.4 (1.5)	2.6 (1.3)	<0.001	3.0 (1.8)	3.2 (1.9)	3.0 (1.9)	0.98
Number of children <14 years	0.4 (0.8)	0.9 (0.9)	1.1 (1.0)	<0.001	1.0 (1.3)	1.1 (1.3)	1.1 (1.2)	0.08
Job hours per day	8.2 (1.2)	8.1 (1.4)	8.1 (1.6)	0.05	6.6 (1.2)	6.9 (1.6)	7.2 (1.8)	<0.001
Commuting time to work, min	47.3 (25.0)	44.7 (23.3)	43.4 (21.6)	0.01	28.0 (20.9)	30.9 (21.8)	33.1 (22.5)	0.001
Regular daytime work, %	75.9	76.0	73.1	0.01	92.2	81.0	70.8	<0.001
Working overtime/Extra job, %	9.9	13.7	19.6	<0.001	8.5	14.4	22.2	<0.001
Current smoker, %	2.1	2.0	2.1	0.07	0.4	0.4	0.0	0.10
Current alcohol drinker, %	68.3	64.9	64.6	0.52	NA	NA	NAO	NA
Physical activity, metabolic equivalent (METs) units/week	55.1 (10.1)	54.3 (9.3)	54.7 (9.0)	0.15	47.5 (9.5)	47.4 (10.1)	47.7 (10.2)	0.77
History of chronic disease, %	9.6	14.8	11.3	0.50	35.1	38.4	38.4	0.007
**Men**								
Number of subjects, *n*	2,073	808	338		786	423	286	
Total work-family conflicts score	1.8 (1.5)	5.9 (1.2)	9.3 (1.6)	<0.001	3.5 (2.1)	7.9 (1.2)	11.6 (1.8)	<0.001
Work-to-family conflict score	1.2 (1.3)	2.9 (1.1)	4.4 (1.4)	<0.001	1.9 (1.5)	3.6 (1.5)	6.4 (1.3)	<0.001
Family-to-work conflict score	0.6 (1.0)	3.0 (1.2)	4.9 (1.6)	<0.001	1.3 (1.3)	3.3 (1.6)	5.2 (1.6)	<0.001
Age, years	43.9 (11.6)	45.3 (9.1)	42.8 (9.2)	0.87	45.0 (10.1)	42.2 (10.1)	40.2 (10.3)	<0.001
High education, %	86.6	87.0	90.1	0.14	49.4	49.4	57.0	0.66
Professional occupation, %	43.6	42.0	48.0	0.39	48.0	46.7	49.0	0.65
Married, %	69.0	85.0	88.9	<0.001	92.0	90.3	86.0	0.75
Living alone, %	9.9	3.4	3.5	<0.001	0.6	1.2	0.7	0.73
Number of family members	2.3 (1.4)	2.6 (1.3)	2.6 (1.3)	<0.001	4.0 (1.8)	3.9 (1.7)	3.7 (1.9)	0.05
Number of children <14 years	0.5 (0.8)	0.9 (1.0)	1.1 (1.0)	<0.001	1.3 (1.4)	1.5 (1.3)	1.4 (1.4)	0.04
Job hours per day	8.3 (1.0)	8.5 (1.2)	8.6 (1.5)	<0.001	7.3 (1.6)	7.7 (1.9)	7.6 (1.9)	<0.001
Commuting time to work, min	56.4 (26.5)	55.6 (25.2)	58.3 (26.4)	0.50	30.5 (20.3)	31.8 (22.6)	31.9 (20.7)	0.25
Regular daytime work, %	93.3	89.5	82.8	<0.001	81.7	75.7	70.3	<0.001
Working overtime/Extra job, %	10.4	17.9	30.4	<0.001	35.3	45.3	69.6	<0.001
Current smoker, %	12.6	11.9	12.6	0.97	10.7	4.3	12.6	0.19
Current alcohol drinker, %	83.1	83.9	79.2	0.26	NA	NA	NA	NA
Physical activity, METs units/week	54.0 (8.8)	54.5 (8.8)	54.7 (8.9)	0.15	48.4 (9.8)	48.8 (9.8)	50.1 (10.2)	0.02
History of chronic disease, %	27.7	27.2	25.2	0.96	30.0	31.8	39.9	<0.001

MET, metabolic equivalent of task; SD, standard deviation.

^a^Continuous variables were expressed as means (SDs) and categorical variables as percentages.

^b^*P*-trend were calculated using age-adjusted linear regression for continuous variables and age-adjusted logistic regression for categorical variables.

Having depression and a low ikigai were observed in 39.4% and 29.9% of Japanese female civil workers and 26.8% and 29.9% of Japanese male civil workers, respectively, while the respective prevalence in Egyptian females were 58.2% and 75.3% and those in Egyptian male civil workers were 38.9% and 63.1%. In a dose-response pattern, levels of TWFCs (Table 2) and its two forms, FWC and WFC (eTable 1), were associated with having depression and a low ikigai in both genders of both countries (*P*-trend <0.0001). After adjusting for a wide range of potential confounders, the final model multivariable ORs of having depression for high versus low levels of TWFCs were 4.11 (95% CI, 2.99–5.65) in Japanese women, 5.42 (95% CI, 4.18–7.02) in Japanese men, 4.43 (95% CI, 3.30–5.65) in Egyptian women, and 4.79 (95% CI, 3.53–6.48) in Egyptian men. The respective ORs of having high ikigai were 0.46 (95% CI, 0.33–0.64) and 0.40 (95% CI, 0.31–0.52) in Japanese women and men, and 0.34 (95% CI, 0.24–0.48) and 0.28 (95% CI, 0.20–0.39) in Egyptian women and men. The ORs of having depression for Egyptian civil workers in reference to Japanese counterparts were 2.63 (95% CI, 2.02–3.44) in women and 2.85 (95% CI, 2.43–3.33) in men, and the ORs of having high ikigai comparing Egyptians to Japanese were 0.22 (95% CI, 0.08–0.15) in women and 0.24 (95% CI, 0.18–0.32) in men (data not shown in tables). However, no significant interactions between TWFCs and the country of the sample were found towards the associations with having depression and a high-ikigai (*P* > 0.05; Table 2).

**Table 2.** Odds ratios and 95% confidence intervals (CIs) for depression and ikigai in Japanese and Egyptian civil workers according to sex- and country-specific categories of total work-family conflicts

	Japan				Egypt			

	Case/total	Model 1	Model 2	Model 3	Case/total	Model 1	Model 2	Model 3
**Depression**								
**Women**	620/1,573				940/1,614			
***TWFCs***								
Low	281/851	1.00	1.00	1.00	321/752	1.00	1.00	1.00
Moderate	189/440	2.09 (1.62–2.70)	2.34 (1.79–3.05)	2.33 (1.78–3.05)	324/479	2.76 (2.16–3.52)	2.82 (2.20–3.61)	2.74 (2.13–3.51)
High	150/282	3.25 (2.42–4.37)	4.07 (2.97–5.58)	4.11 (2.99–5.65)	295/383	4.28 (3.22–5.68)	4.36 (3.26–5.84)	4.43 (3.30–5.95)
*P*-trend		<0.0001	<0.0001	<0.0001		<0.0001	<0.0001	<0.0001
**Men**	864/3,219				581/1,495			
***TWFCs***								
Low	408/2,073	1.00	1.00	1.00	187/786	1.00	1.00	1.00
Moderate	281/808	2.64 (2.19–3.19)	2.70 (2.22–3.27)	2.72 (2.23–3.30)	213/423	3.14 (2.44–4.06)	3.12 (2.41–4.03)	3.10 (2.40–4.01)
High	175/338	5.44 (4.24–6.98)	5.45 (4.21–7.06)	5.42 (4.18–7.02)	181/286	5.18 (3.85–6.96)	4.92 (3.64–6.64)	4.79 (3.53–6.48)
*P*-trend		<0.0001	<0.0001	<0.0001		<0.0001	<0.0001	<0.0001
**Ikigai**								
**Women**	1,102/1,753				399/1,614			
***TWFCs***								
Low	614/851	1.00	1.00	1.00	240/752	1.00	1.00	1.00
Moderate	308/440	0.74 (0.57–0.87)	0.72 (0.54–0.85)	0.70 (0.53–0.83)	103/479	0.55 (0.42–0.73)	0.56 (0.43–0.74)	0.57 (0.43–0.75)
High	180/282	0.53 (0.39–0.72)	0.48 (0.34–0.66)	0.46 (0.33–0.64)	56/383	0.33 (0.24–0.46)	0.34 (0.24–0.47)	0.34 (0.24–0.48)
*P*-trend		<0.0001	<0.0001	<0.0001		<0.0001	<0.0001	<0.0001
**Men**	2,256/3,219				552/1,495			
***TWFCs***								
Low	1,509/2,073	1.00	1.00	1.00	369/786	1.00	1.00	1.00
Moderate	544/808	0.55 (0.44–0.78)	0.53 (0.42–0.76)	0.53 (0.42–0.76)	126/423	0.45 (0.35–0.58)	0.46 (0.36–0.59)	0.45 (0.35–0.59)
High	203/338	0.42 (0.33–0.54)	0.40 (0.31–0.52)	0.40 (0.31–0.52)	57/286	0.26 (0.19–0.37)	0.28 (0.20–0.38)	0.28 (0.20–0.39)
*P*-trend		<0.0001	<0.0001	<0.0001		<0.0001	<0.0001	<0.0001

TWFCs, total work-family conflicts.

Model 1: Adjusted for sociodemographic factors (age, education, occupation, and marital status).

Model 2: Adjusted further for family factors (family structure, number of family members, and the presence of family members below 14 years old), and work factors (job hours per day, time to commute to work, shift work, and working overtime or extra job).

Model 3: Adjusted further for behavioral factors (smoking status and physical activity), and alcohol drinking status in the Japanese cohort, and history of chronic diseases (hypertension, diabetes, dyslipidemia, ischemic heart disease, stroke, kidney diseases, liver diseases, and cancer), country, and the interaction of TWFCs by country.

*P*-interaction between TWFCs and country of sample origin in Model 3 were 0.69 in women and 0.46 in men for depression and 0.26 in women and 0.38 in men for ikigai.

The observed associations did not differ in stratified analyses by family structure (eTable 2), shift work, or occupational class (eTable 3). Similarly, stratification by age (<40 vs ≥40 years), marital status (married vs others), number of junior family members below 14 years old (<2 vs ≥2), working overtime or extra work (yes vs no), job hours per day (<sex- and country-specific median value vs ≥median value), and having a history of chronic diseases (yes vs no) did not alter the observed associations (data not shown in tables).

One-point increment of the scores of TWFCs, FWC, and WFC was associated with 0.25–0.89-point increment in the CES-D score in Japanese and Egyptian male and female civil workers (*P* < 0.0001; Table 3). The ikigai level slightly mediated the observed slobs for the CES-D score by the conflict scores. The mediation percentage was higher in Egyptian women and men than in Japanese counterparts and was higher for WFC than for FWC.

**Table 3.** Mediation analyses for the ikigai (as a mediator) on the pathway between work-family conflicts scores (as exposure) and the Center for Epidemiological Studies Depression (CES-D) score (as outcome)

	Total effect		Direct effect		Indirect effect		Percentage mediated

	Effect size (95% CI)	*P*-value	Effect size (95% CI)	*P*-value	Effect size (95% CI)	*P*-value	
**TWFCs**							
Japanese women	0.25 (0.21–0.30)	<0.0001	0.24 (0.19–0.29)	<0.0001	0.01 (0.003–0.02)	<0.0001	4.6%
Japanese men	0.33 (0.29–0.36)	<0.0001	0.31 (0.27–0.34)	<0.0001	0.02 (0.01–0.03)	<0.0001	6.6%
Egyptian women	0.54 (0.48–0.60)	<0.0001	0.46 (0.40–0.52)	<0.0001	0.08 (0.06–0.10)	<0.0001	14.4%
Egyptian men	0.54 (0.49–0.60)	<0.0001	0.49 (0.43–0.55)	<0.0001	0.05 (0.03–0.07)	<0.0001	9.4%
**FWC**							
Japanese women	0.25 (0.17–0.32)	<0.0001	0.24 (0.17–0.32)	<0.0001	0.03 (−0.02–0.02)	0.48	1.0%
Japanese men	0.36 (0.30–0.42)	<0.0001	0.34 (0.28–0.39)	<0.0001	0.02 (0.01–0.04)	<0.0001	8.1%
Egyptian women	0.89 (0.77–1.00)	<0.0001	0.76 (0.64–0.87)	<0.0001	0.13 (0.09–0.17)	<0.0001	14.5%
Egyptian men	0.87 (0.76–0.98)	<0.0001	0.78 (0.67–0.89)	<0.0001	0.09 (0.06–0.12)	<0.0001	10.0%
**WFC**							
Japanese women	0.54 (0.45–0.64)	<0.0001	0.50 (0.41–0.60)	<0.0001	0.04 (0.02–0.06)	<0.0001	6.5%
Japanese men	0.62 (0.55–0.68)	<0.0001	0.58 (0.51–0.64)	<0.0001	0.04 (0.03–0.06)	<0.0001	6.7%
Egyptian women	0.67 (0.57–0.77)	<0.0001	0.55 (0.45–0.65)	<0.0001	0.12 (0.09–0.16)	<0.0001	18.0%
Egyptian men	0.75 (0.66–0.85)	<0.0001	0.66 (0.56–0.76)	<0.0001	0.10 (0.06–0.13)	<0.0001	12.7%

FWC, family-to-work conflict; TWFCs, total work-family conflicts; WFC, work-to-family conflict.

Outcome (CES-D score) and mediator (ikigai level) modelled by beta slope of linear regression models and the total, direct, and indirect effects (bootstrap = 5,000 times) determined using PROCESS macro (Hayes, 2013). The effect size is the adjusted for age, education, occupation, marital status, family structure, number of family members, presence of family members below 14 years old, job hours per day, time to commute to work, shift work, working overtime or extra job, smoking status, physical activity, alcohol drinking status in the Japanese cohort, history of hypertension, diabetes, dyslipidemia, ischemic heart disease, stroke, kidney diseases, liver diseases, and cancer.

## DISCUSSION

In this cross-cultural study, higher levels of TWFCs were positively associated with the prevalence of having depression and inversely associated with the prevalence of having a high ikigai in a dose-response pattern among Japanese and Egyptian civil workers. The observed associations were independent of a wide range of hypothesized confounding factors and did not vary by different levels of these factors. The level of ikigai slightly mediated the observed associations. The mediation percentage was higher in Egyptian than Japanese civil workers and for the association of WFC rather than FWC with the CES-D score.

The positive association between TWFCs and having depression found in this research was consistent with the findings from previous studies conducted in different working populations, including the American,^11^^–^^14^ Finish,^15^ British,^15^ Swiss,^16^ and Malaysian^17^ employees, despite the different cultural situations beyond the epidemiology field. In this regard, Japan and Egypt have some similarities and differences in work and family culture. For example, at the work level, the lifetime-employment system in both countries is one similarity; yet, the longer working hours for Japanese civil workers than Egyptians is one of the differences. According to the 2016 Japan Social Life Basic Survey, the average commuting time for Japanese workers aged 15 years and older was 84 minutes for men and 66 minutes for women, and the respective average daily working hours were 9.9 hours and 7.9 hours on the day of the survey.^32^ On the other hand, according to Egypt’s Labor Market Panel Survey (wave of 2012), the average estimated commuting time to work was 32 minutes, and the average daily working hours was 6.4 hours.^33^ The male breadwinner and female caregiver image were similar, but there were larger family structure and number of children per family for the Egyptian than the Japanese civil workers.

In a comparative study between Japan, the United Kingdom, and Finland, Chandola et al showed that TWFCs mediated the effect of having multiple roles (playing different roles for family and work simultaneously) on the men and women’s mental health in the three countries.^15^ In our comparative study, the observed association between TWFCs and psychological health was stronger in Egyptian compared to Japanese civil workers. However, it did not vary by gender or factors related to the participants’ age, work environment, and family attributes. Similar to our results, Wang et al did not find any effect modifications by age, gender, working hours, or domestic roles on the association between TWFCs and mental health status among 4,553 American working participants in the National Comorbidity Survey.^13^

Our study is the first study to investigate the association between TWFCs and having ikigai in Japanese and non-Japanese populations. Previously, TWFCs were shown associated with several health outcomes, mainly life satisfaction.^34^ One aspect of the ikigai concept is to feel satisfied with your life^20^; accordingly, the observed inverse associations between TWFCs and having ikigai were expected. Despite being a Japanese concept, the ikigai purpose of life was tested among other populations^35^^,^^36^ including Egyptians^36^ and showed good internal reliability and similar associations with psychological well-being. The lower prevalence of having high ikigai reported by the Egyptian (24.7% in women and 36.9% in men) than Japanese civil workers (70.1% in women and men) could be partially attributed to the fact that the ikigai concept itself was new to the Egyptian civil workers. With the detailed information of the ikigai question in the Egyptian survey, many participants refrained from the higher self-report levels to that questions.

Moreover, the current study is the first to investigate the hypothesis of mediation by ikigai level on the association of the conflicts between work and family with psychological health. Our results suggested that having low ikigai mediated the associations between TWFCs and depression for Japanese and Egyptian women and men. This new finding added to the current knowledge about pathways by which the TWFCs affect the psychological health of civil workers. The different percentage mediation observed in men and women suggests that the mediating effect could vary by gender and population.

Mechanisms by which TWFCs may associate with adverse mental health outcomes such as depression, included the development of life stress,^11^ decreased physical health,^26^^,^^27^ and diminished emotional well-being,^17^ with which subjects cannot be happy or enjoy their life (low ikigai)^21^^,^^22^ and could proceed to psychological distress^19^ and feeling the loss of resources,^15^^,^^28^ resulting in a poor mental health status.^12^^,^^16^

The relatively large sample size of civil workers from two different populations that allowed several stratified analyses and gave the power to conduct sex- and country-specific mediation analysis is one of the strengths of the current study. Also, we used a validated TWFCs expanded algorithm rather than one assessment question. However, our study is not without limitations. The causal inference was impossible using cross-sectional data; yet, the observed consistent associations could indicate a potential cause-effect relationship, especially since we have adjusted the statistical models for a wide range of potential confounders and stratified by gender and many other factors. Second, the generalizability of the study findings is questionable, since the recruited participants were civil workers; moreover, the volunteer nature of the convenient sampling used in the Egyptian survey let the proportion of female respondents be higher than males, which should not be interpreted as higher proportions of female Egyptian civil workers than male ones. Also, there was an overrepresentation of highly educated and professional civil workers in both the Egyptian and Japanese samples. Third, several sources of bias could accompany the collection of self-reported data; however, we used the validated CES-D tool^29^^,^^37^ to assess the outcome variable and proper scoring algorithms to assess the exposure variables that showed high reliability in both Egyptian and Japanese populations.^19^^,^^26^ Even though the CES-D score provided reasonable and meaningful within-individual variation related to their mood disorders in both the Egyptian and Japanese surveys, the fact that we did not validate the questionnaire in our populations, especially in terms of the cutoff point, is a limitation worth mentioning. Kohout et al explained that it is appropriate to lower or raise the used cutoff point to guard against false positives or negatives as it suits the study’s population.^38^ We tested the associations using different cutoff points indicating depression; ≥7 as Kohout et al,^38^ ≥8 as Ota et al,^30^ ≥9 as Torres,^37^ and ≥10 points of the CES-D score, and the associations did not change materially. Last, despite the explicit explanation given to the Egyptian participants regarding the meaning of the ikigai question, a gap might still be present between the Japanese and Egyptian understanding of the ikigai concept. Further studies are needed to investigate the different ikigai perceptions by Japanese and non-Japanese populations and their impact on mediating health outcomes.

In conclusion, TWFCs showed positive associations with having depression in a dose-response pattern in both genders of Japanese and Egyptian civil workers. These conflicts were inversely associated with having ikigai, which mediated the observed associations between TWFCs and psychological health. These findings imply that improving ikigai may be a possible pathway for addressing the mechanism connecting TWFCs to adverse psychological health outcomes. Authorities in both Japan and Egypt should aim to alleviate TWFCs of civil workers, which could improve organizational productivity and the familial context of the society by supporting the favorable psychological health of the civil workers.

## SUPPLEMENTARY MATERIAL

eTable 1. Multivariable odds ratios and 95% confidence intervals for depression and ikigai in Japanese and Egyptian civil workers according to sex- and country-specific categories of work-to-family conflict and family-to-work conflict

eTable 2. Stratified analyses by family structure for depression and ikigai in Japanese and Egyptian civil workers according to categories of total work-family conflicts

eTable 3. Stratified analyses by work schedule and occupational class for depression and ikigai in Japanese and Egyptian civil workers according to categories of total work-family conflicts
