# Supplementary material for: Work and Family Conflicts, Depression, and “Ikigai”: A Mediation Analysis in a Cross-cultural Study Between Japanese and Egyptian Civil Workers
Source: J Epidemiol. 2023 Jul 5;33(7):360–6. doi: 10.2188/jea.JE20210338 (PMC10257991; doi:10.2188/jea.JE20210338)
Supplement: Supplementary file 1 [file je-33-360-s001.pdf]

eTable 1. Multivariable odds ratios and 95% confidence intervals for depression and ikigai in Japanese and Egyptian civil workers according to sex- and country-specific categories of work-to-family conflict and family-to-work conflict

	Japan				Egypt			
	FWC		WFC		FWC		WFC	
	Case/total	OR (95% CI)[a]	Case/total	OR (95% CI)[a]	Case/total	OR (95% CI)[a]	Case/total	OR (95% CI)[a]
**Depression**								
**Women**	620/1,573		620/,1573		940/1,614		940/1,614	
Low	262/739	1.00	163/555	1.00	303/690	1.00	239/557	1.00
Moderate	236/598	1.97 (1.52–2.56)	345/827	2.12 (1.65–2.72)	473/717	2.57 (2.05–3.21)	399/664	1.89 (1.49–2.39)
High	122/236	4.00 (2.81–5.68)	112/191	5.13 (3.52–7.49)	164/207	5.17 (3.54–7.57)	302/393	4.14 (3.04–5.64)
P-trend		<0.0001		<0.0001		<0.0001		<0.0001
**Men**	864/3,219		846/3,219		581/1,495		581/1,495	
Low	380/1,846	1.00	226/1410	1.00	192/772	1.00	126/562	1.00
Moderate	368/1,122	2.51 (2.09–3.03)	493/1566	2.87 (2.37–3.48)	288/576	2.91 (2.29–3.70)	257/591	2.52 (1.94–3.28)
High	116/251	4.16 (3.10–5.58)	145/243	9.30 (6.76–12.80)	101/147	6.43 (4.33–9.56)	198/342	4.09 (3.00–5.57)
P-trend		<0.0001		<0.0001				<0.0001
**Ikigai**								
**Women**	1102/1,573		1102/1,573		399/1,614		399/1,614	
Low	520/739	1.00	411/555	1.00	218/690	1.00	194/557	1.00
Moderate	423/598	0.76 (0.59–0.92)	573/827	0.71 (0.55–0.82)	147/717	0.53 (0.41–0.68)	151/664	0.54 (0.42–0.70)
High	159/236	0.56 (0.39–0.71)	118/191	0.44 (0.30–56)	34/207	0.40 (0.27–0.61)	54/393	0.28 (0.19–0.39)
P-trend		<0.0001		<0.0001		<0.0001		<0.0001
**Men**	2256/3,216		2,256/3,219		552/1,495		552/1,495	
Low	1339/1,846	1.00	1,044/1,410	1.00	337/772	1.00	287/562	1.00
Moderate	773/1,122	0.64 (0.54–0.76)	1,071/1,566	0.65 (0.54–0.77)	182/576	0.59 (0.47–0.75)	199/591	0.45 (0.35–0.57)
High	144/251	0.34 (0.26–0.46)	141/243	0.38 (0.28–0.52)	33/147	0.36 (0.24–0.55)	66/342	0.22 (0.16–0.31)
P-trend		<0.0001		<0.0001		<0.0001		<0.0001

CI, confidence interval; FWC, family-to-work conflict; OR, odds ratio; WFC, work-to-family conflict.

[a] Adjusted for age, education, occupation, marital status, family structure, number of family members, the presence of family members below 14 years old, job hours per day, time to commute to work, shift work, working overtime or extra job, smoking status and physical activity, alcohol drinking status in the Japanese cohort, histories of hypertension, diabetes, dyslipidemia, ischemic heart disease, stroke, kidney diseases, liver diseases, and cancer, country, and the interaction of total work-family conflicts by country.

P-interaction between FWC and country of sample origin were 0.28 in women and 0.20 in men for depression and were 0.11 in women and 0.81 in men for ikigai. The respective p-interactions between WFC and country of sample origin were 0.66 in women and 0.36 in men for depression and were 0.15 in women and 0.51 in men for ikigai.

eTable 2. Stratified analyses by family structure for depression and ikigai in Japanese and Egyptian civil workers according to categories of total work-family conflicts

	Japan			Egypt		
	**Couple**	**Nuclear family**	**multigeneration family and others**	**Couple**	**Nuclear family**	**multigeneration family and others**
	OR (95% CI)[a]	OR (95% CI)[a]	OR (95% CI)[a]	OR (95% CI)[a]	OR (95% CI)[a]	OR (95% CI)[a]
**Depression**						
**Women**						
Low	1.00	1.00	1.00	1.00	1.00	1.00
Moderate	2.96 (1.52–5.77)	1.81 (1.01–3.59)	2.75 (1.88–4.03)	3.53 (1.46–8.53)	2.16 (1.44–3.22)	3.72 (2.51–5.50)
High	6.15 (2.12–17.83)	3.99 (2.00–7.97)	4.56 (2.79–7.45)	8.08 (2.98–23.35)	3.36 (2.08–5.41)	5.85 (3.57–9.53)
P-trend	<0.0001	<0.0001	<0.0001	<0.0001	<0.0001	<0.0001
**Men**						
Low	1.00	1.00	1.00	1.00	1.00	1.00
Moderate	2.10 (1.25–3.55)	1.96 (1.35–2.84)	3.73 (2.82–4.94)	2.67 (1.84–3.88)	4.31 (2.79–6.67)	2.67 (1.84–3.88)
High	3.14 (1.51–6.53)	3.69 (2.42–5.62)	10.45 (6.68–16.34)	4.77 (3.04–7.48)	6.89 (3.94–12.03)	4.77 (3.04–7.48)
P-trend	<0.0001	<0.0001	<0.0001	<0.0001	<0.0001	<0.0001
**Ikigai**						
**Women**						
Low	1.00	1.00	1.00	1.00	1.00	1.00
Moderate	0.29 (0.10–0.85)	0.97 (0.41–2.31)	0.61 (0.42–0.88)	0.66 (0.25–0.88)	0.79 (0.51–1.23)	0.42 (0.27–0.65)
High	0.29 (0.14–0.61)	0.56 (0.23–1.34)	0.25 (0.15–0.40)	0.35 (0.12–0.72)	0.30 (0.16–0.56)	0.41 (0.25–0.68)
P-trend	<0.0001	0.17	<0.0001	0.001	0.03	<0.0001
**Men**						
Low	1.00	1.00	1.00	1.00	1.00	1.00
Moderate	0.64 (0.38–0.97)	0.71 (0.48–1.06)	0.51 (0.39–0.66)	0.16 (0.03–0.57)	0.40 (0.23–0.68)	0.58 (0.36–0.82)
High	0.29 (0.14–0.61)	0.40 (0.25–0.62)	0.29 (0.19–0.45)	0.03 (0.01–0.22)	0.27 (0.18–0.43)	0.32 (0.19–0.53)
P-trend	<0.0001	<0.0001	<0.0001	<0.0001	<0.0001	<0.0001

CI, confidence interval; OR, odds ratio.

[a] Adjusted for age, education, occupation, marital status, number of family members, the presence of family members below 14 years old, job hours per day, time to commute to work, shift work, working overtime or extra job, smoking status and physical activity, alcohol drinking status in the Japanese cohort, and history of hypertension, diabetes, dyslipidemia, ischemic heart disease, stroke, kidney diseases, liver diseases, and cancer.

eTable 3. Stratified analyses by work schedule and occupational class for depression and ikigai in Japanese and Egyptian civil workers according to categories of total work-family conflicts

	Japan				Egypt			
	**Work schedule**		**Occupational Class**		**Work schedule**		**Occupational Class**	
	Regular day time work	***Shift work***	***Professional***	***Other than professional***	***Regular day time work***	***Shift work***	***Professional***	***Other than professional***
	OR (95% CI)[a]	OR (95% CI)[a]	OR (95% CI)[a]	OR (95% CI)[a]	OR (95% CI)[a]	OR (95% CI)[a]	OR (95% CI)[a]	OR (95% CI)[a]
**Depression**								
**Women**								
Low	1.00	1.00	1.00	1.00	1.00	1.00	1.00	1.00
Moderate	2.30 (1.66–3.19)	3.67 (1.85–7.31)	2.65 (1.77–3.97)	2.29 (1.50–3.49)	2.67 (2.03–3.52)	3.83 (1.51–9.70)	2.79 (2.03–3.84)	2.81 (1.79–4.42)
High	4.25 (2.86–6.32)	8.70 (3.75–20.20)	4.89 (3.03–7.88)	4.82 (2.79–8.33)	5.16 (3.64–7.31)	3.85 (1.59–9.46)	4.85 (3.31–7.09)	4.42 (2.57–7.59)
P-trend	<0.0001	<0.0001	<0.0001	<0.0001	<0.0001	<0.0001	<0.0001	<0.0001
**Men**								
Low	1.00	1.00	1.00	1.00	1.00	1.00	1.00	1.00
Moderate	2.75 (2.23–3.39)	5.27 (2.33–11.91)	2.76 (2.02–3.77)	2.86 (2.18–3.75)	2.67 (1.97–3.61)	3.98 (2.15–7.36)	3.33 (2.25–4.93)	2.85 (1.96–4.15)
High	5.28 (3.96–7.05)	23.00 (8.18–64.67)	8.59 (5.71–12.91)	4.46 (3.06–6.49)	5.64 (3.89–8.47)	4.98 (2.34–10.61)	6.27 (3.85–9.22)	4.85 (3.08–7.63)
P-trend	<0.0001	<0.0001	<0.0001	<0.0001	<0.0001	<0.0001	<0.0001	<0.0001
**Ikigai**								
**Women**								
Low	1.00	1.00	1.00	1.00	1.00	1.00	1.00	1.00
Moderate	0.53 (0.38–0.75)	0.80 (0.40–1.63)	0.50 (0.33–0.78)	0.62 (0.40–0.97)	0.66 (0.49–0.90)	0.39 (0.14–0.96)	0.54 (0.39–0.77)	0.76 (0.46–1.03)
High	0.33 (0.21–0.51)	0.22 (0.10–0.49)	0.30 (0.18–0.51)	0.30 (0.17–0.54)	0.39 (0.26–0.58)	0.36 (0.13–0.67)	0.31 (0.20–0.49)	0.53 (0.29–0.86)
P-trend	<0.0001	0.0005	<0.0001	<0.0001	<0.0001	<0.0001	<0.0001	0.002
**Men**								
Low	1.00	1.00	1.00	1.00	1.00	1.00	1.00	1.00
Moderate	0.60 (0.49–0.74)	0.46 (0.18–1.21)	0.60 (0.44–0.82)	0.58 (0.45–0.75)	0.52 (0.38–0.70)	0.53 (0.29–0.88)	0.36 (0.24–0.54)	0.62 (0.43–0.89)
High	0.30 (0.22–0.40)	0.39 (0.18–0.84)	0.37 (0.25–0.56)	0.27 (0.18–0.40)	0.27 (0.18–0.41)	0.41 (0.19–0.79)	0.29 (0.17–0.49)	0.28 (0.17–0.47)
P-trend	<0.0001	0.0004	<0.0001	<0.0001	<0.0001	0.003	<0.0001	<0.0001

CI, confidence interval; OR, odds ratio.

[a] Adjusted for age, education, occupation (for shift work stratification), marital status, family structure, number of family members, the presence of family members below 14 years old, job hours per day, time to commute to work, shift work (for occupational class stratification), working overtime or extra job, smoking status and physical activity, alcohol drinking status in the Japanese cohort, and history of hypertension, diabetes, dyslipidemia, ischemic heart disease, stroke, kidney diseases, liver diseases, and cancer.
